# Reduction of microRNA-221 in BVDV infection enhances viral replication by targeting the ATG7-mediated autophagy pathway

**DOI:** 10.1186/s13620-025-00286-3

**Published:** 2025-04-02

**Authors:** Zihan Chen, Jingyu Wang, Baochun Lu, Wenxin Meng, Yufan Zhu, Qifeng Jiang, Duo Gao, Zihang Ma, Huijuan Zeng, Jinping Chen, Shizhe Liu, Zhen Wang, Kun Jia

**Affiliations:** 1 College of Veterinary Medicine, South China Agricultural University, Guangdong, 510642 China; 2 College of Veterinary Medicine, Jilin University, Changchun, 130062 China

**Keywords:** Bovine viral Diarrhoea virus, ATG7-LC3, MicroRNA, Cellular autophagy, Viral replication

## Abstract

**Background:**

Bovine viral diarrhoea (BVD), a condition triggered by bovine viral diarrhoea virus (BVDV), is recognized globally as a prevalent pathogen among ruminants and markedly affects the economics of animal husbandry. MicroRNAs, a class of small noncoding RNAs, play pivotal roles in regulating a myriad of biological processes.The ATG7-LC3 pathway, a canonical autophagy mechanism, is integral in defending against pathogenic invasion and maintaining cellular homeostasis.

**Results:**

In this study, we observed significant downregulation of bta-miR-221 in cells infected with BVDV. We further established that overexpression of bta-miR-221 markedly attenuated BVDV replication in Madin‒Darby bovine kidney (MDBK) cells. Through bioinformatics prediction analysis, we identified ATG7, an autophagy-related gene, as a direct downstream target of bta-miR-221. However, the intricate relationships among bta-miR-221, the ATG7-LC3 pathway, and BVDV infection remained unclear. Our study revealed that ATG7 expression was significantly elevated in BVDV-infected cells, whereas bta-miR-221 mimics repressed both endogenous and exogenous ATG7 expression. Following BVDV infection, we noted a decrease in LC3I expression, its conversion to LC3II, a significant increase in ATG7 expression, and a notable decrease in SQSTM1/p62 expression. By employing laser confocal microscopy and immunoprecipitation assays, we elucidated the regulation of the ATG7-LC3 pathway by bta-miR-221 in MDBK cells. Our findings recealed that BVDV infection enhanced the ATG7-LC3 interaction, inducing autophagy through the suppression of bta-miR-221 in MDBK cells. Consequently, bta-miR-221 emerged as a potent inhibitor of BVDV, impacting its proliferation and replication within the host.

**Conclusions:**

This research sheds light on novel aspects of virus-host interactions and lays a foundation for the development of antiviral therapeutics.

## Introduction

Bovine viral diarrhoea virus (BVDV), a pivotal pathogen implicated in bovine viral diarrhoea (BVD), is classified within the genus Pestivirus of the family Flaviviridae [1]. BVDV poses a significant challenge to livestock farming worldwide, critically impacting the economic landscape of the industry [2]. The most pernicious impact of this pathogen arises from its ability to establish persistent infections (PIs) in cattle, which serve as reservoirs, continuously shedding the virus throughout their lifespan and facilitating its spread within herds [3]. In addition to its identification in bovine populations, BVDV has been detected across a spectrum of domestic [4–6] and wild animal species [7, 8]. Infection during pregnancy in sheep, goats, pigs, and wild ruminants leads to clinical manifestations analogous to those observed in cattle [9]. The copresence and interaction of these species, both domesticated and wild, create an environment that is conducive to interspecies viral transmission, underscoring the broader implications of BVDV epidemiology and control strategies. Despite its importance, the molecular intricacies of BVDV-host interactions remain largely elusive.

**Fig. 1:**
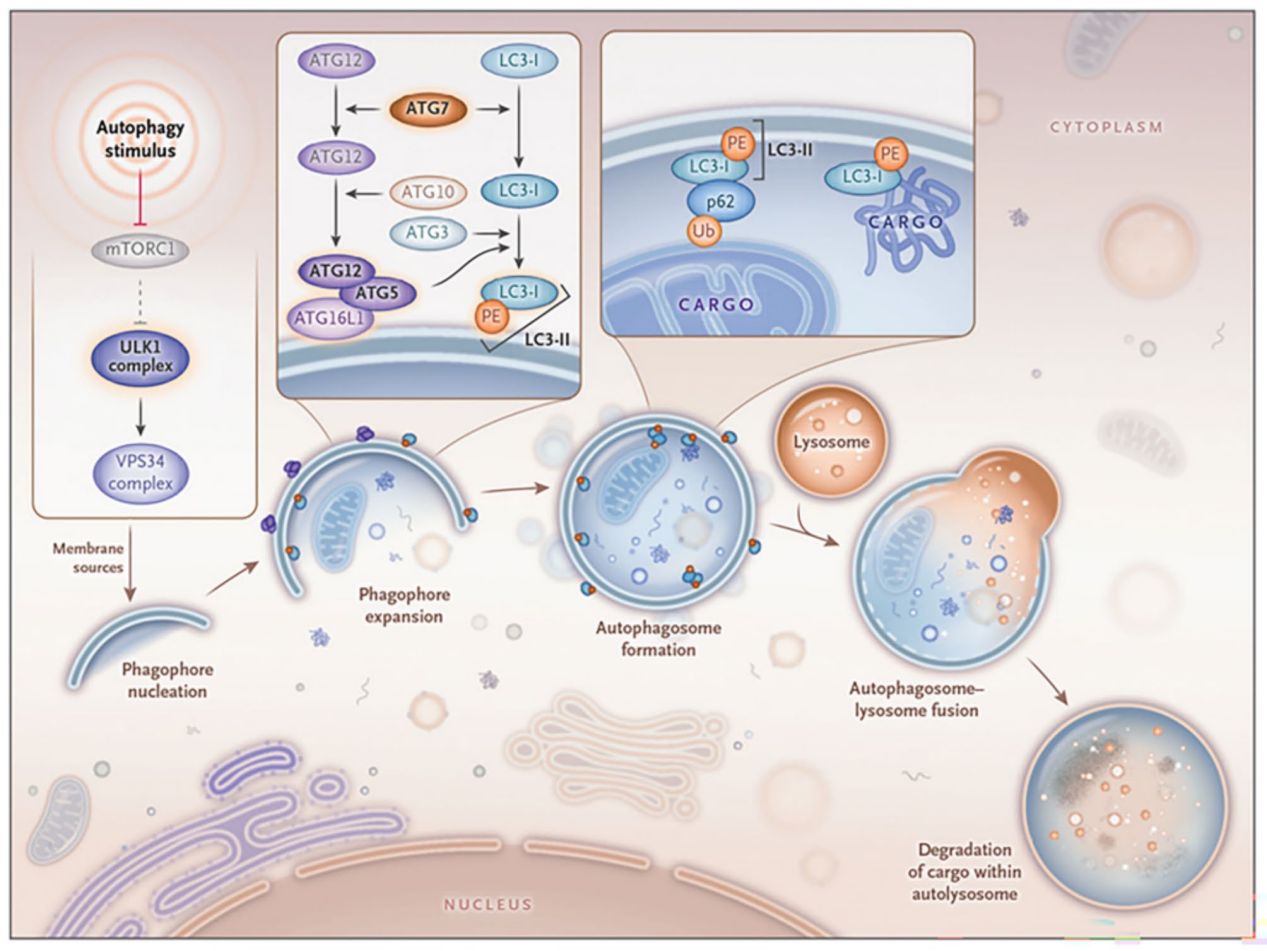
Classical Degradative Autophagy [18]. The depicted schematic elucidates the pivotal role of the Unc-51-like kinase 1 (ULK1) complex in orchestrating a spectrum of upstream signals, culminating in the initiation of an autophagic cascade. This process involves the synthesis of ATG5-ATG12 and ATG8 (LC3 II), which, under the catalytic influence of ATG7, coalesce to assemble autophagosomes. Concurrently, cellular constituents exert their influence via a dualistic engagement mechanism: a direct liaison with the LC3 protein and an indirect association mediated by the p62 receptor

Autophagy, a sophisticated cellular mechanism, involves the transport of intracellular components to lysosomes for degradation and recycling, with macroautophagy being the predominant form [10]. This process, hereafter referred to as autophagy, is a lysosome-dependent degradation pathway in which cytoplasmic constituents are sequestered in double-membrane vesicles, known as autophagosomes [10], and then degraded to bolster organismal survival under diverse environmental stresses [11]. Essential autophagy-related genes (Atgs), such as mTOR, Beclin-1, LC3, and p62, orchestrate the autophagic process [12]. mTOR serves as an upstream regulator of autophagy [13], and Beclin-1 and LC3 are implicated in autophagosome formation, whereas p62 plays a role in their degradation [14, 15]. Notably, LC3 undergoes a ubiquitination-like modification, interacting sequentially with ATG7, ATG3, and the ATG12-ATG5 complex to attach to phosphatidylethanolamine (PE), converting it from a soluble form to a membrane-bound form [16]. Additionally, TP53INP2 has been shown to facilitate LC3 lipidation by interacting with the LC3-ATG7 stabilizing complex [17] (Fig. 1). Given these insights, it is postulated that LC3 and ATG7 interactions play pivotal roles in Madin‒Darby bovine kidney (MDBK) cells, the host cells for BVDV, underscoring a potential mechanistic pathway influencing BVDV pathogenesis and the host cell response.

MicroRNAs (miRNAs), small noncoding RNAs approximately 22 nucleotides in length, play crucial roles in gene regulation by binding to the 3’UTR (untranslated region) of target mRNAs. This interaction leads to mRNA degradation and translational silencing, effectively inhibiting protein production [19]. The regulatory functions of miRNAs extend across various biological processes, including the modulation of viral infection and replication mechanisms. For example, the miRNA let-7 is secreted into the cytoplasm through extracellular vesicles (EVs), where it contributes to the host’s antiviral defences and influences viral replication dynamics [20]. Similarly, miRNA-133a has been identified as a regulator of dengue virus (DENV) replication by targeting host factors that are essential for viral proliferation [21]. Notably, hepatitis C virus (HCV), which shares structural similarities with BVDV, exhibits differential miRNA expression profiles capable of directly or indirectly modulating HCV replication during infection [22]. Given the utility of BVDV as a surrogate model for HCV research [23], it is postulated that miRNAs might similarly influence BVDV replication, underscoring the potential for miRNAs to serve as key regulators in the viral lifecycle and host-virus interactions.

In our research, we elucidated the role of bta-miR-221 as a suppressive agent in the BVDV infection process, revealing how BVDV modulates the ATG7-LC3-mediated autophagy pathway through the manipulation of bta-miR-221 expression. This modulation, in turn, influences cellular autophagic responses. Our findings shed light on the intricate mechanism of interaction between BVDV and host autophagy, offering novel insights into viral pathogenesis. Furthermore, this study paves the way for future research into viral mechanisms and the development of innovative antiviral therapies.

## Materials and methods

### Antibodies and reagents

The LC3B antibody (ab63817) was sourced from Abcam, Shanghai, China. Anti-ATG7 (10088-2-AP) and ant-GAPDH (60004-1-lg) antibodies were obtained from Proteintech, USA. The BVDV E2 antigen was maintained in our laboratory. The SQSTM1/p62 (A11483) antibody was acquired from ABclonal, Wuhan, China, while the GFP (GTX859) and mCherry (GTX128508) antibodies were purchased from GeneTex, USA. Mouse IgG and rabbit IgG fluorescent antibodies (5257P and 5151P, respectively) were procured from Cell Signaling Technology, Shanghai, China. Transfection reagents, including Opti-MEM, were supplied by Gibco, USA. Lipofectamine™ 3000 was purchased from Invitrogen, USA, and Lipo8000 was obtained from Beyotime, Shanghai, China. Additionally, Dulbecco’s modified Eagle’s medium (DMEM), foetal bovine serum (FBS), and phosphate-buffered saline (PBS) were obtained from VivaCell Biosciences, Shanghai, China. This procurement list underscores the diverse range of high-quality reagents and tools utilized in our study, ensuring the reliability and reproducibility of our experimental results.

### Cell and virus cultures

MDBK cells and human embryonic kidney cells (HEK-293T) cells, which were maintained by the Department of Surgery at the College of Veterinary Medicine, South China Agricultural University, were cultured in DMEM supplemented with 10% FBS. The cell cultures were incubated at 37°C in a humidified atmosphere containing 5% CO2. BVDV strains, sourced from Inner Mongolia Agricultural University, were preserved and propagated in MDBK cells within the same department. Viral titres were quantified by calculating the 50% tissue culture infectious dose (TCID50), facilitating the determination of the multiplicity of infection (MOI) for each experiment. Relative viral expression levels were assessed via reverse transcription‒quantitative polymerase chain reaction (RT‒qPCR) with specific primers targeting the BVDV 5’UTR: forward primer 5’-CATGCCCTTAGTAGGACTAGC-3’ and reverse primer 5’-CGAACCACTGACGACTACC-3’. This methodological approach ensured the accurate evaluation of BVDV replication dynamics in cultured cells.

### High-throughput sequencing data analysis

MDBK cells were seeded in six-well plates and categorized into two groups: normal cultured cells (control) and treated cells (treated), which reached a confluency of 60–70% prior to treatment. After treatment, the cells were harvested and stored at -80 °C following a 48-hour incubation period. The miRNA library construction and sequencing analysis were conducted in accordance with the protocols provided by BGI (Shenzhen, 518083, China), utilizing the DNBSEQ platform for sequencing. A single-end 50 (SE50) sequencing strategy was employed, referencing the bovine genome version GCF_000003205.7_Btau_5.0.1. This approach facilitated the comprehensive examination of miRNA expression profiles, enabling the identification of miRNAs potentially involved in the response to the treatment in MDBK cells.

### RNA isolation and quantitative PCR

Following the high-throughput sequencing analysis, twelve miRNAs displaying significant differential expression were selected for validation of their expression levels. Prior to RNA extraction, the cells subjected to 48 h of viral exposure were washed three times with PBS. Total RNA was then extracted using the RNAfast200 Total RNA Extraction Kit (Shanghai Feijie Biotechnology Co., Ltd., China). For miRNA isolation, HiScript III All-in-one RT SuperMix Perfect for qPCR coupled with the miRNA 1st Strand cDNA Synthesis Kit (using stem‒loop methodology) were used according to the manufacturer’s instructions. Reverse transcription and qPCR analyses were conducted using ChamQ Universal SYBR qPCR Master Mix on a LightCycler^®^480 II PCR system (Roche, Basel, Switzerland). Both the reverse transcription and qPCR reagents were obtained from Vazyme Biotech Co. Ltd (Nanjing, China), ensuring the accurate quantification of miRNA expression changes posttreatment. The expression levels of the target genes were determined using the 2^−ΔΔCT^ method, with GAPDH serving as the normalization control. To ensure the robustness and validity of the results, each experimental condition was replicated three times. The sequences of the miRNAs of interest were sourced from the NCBI and miRBase databases, and input into miRNA Design V1.01 software to generate the necessary stem‒loop and primer sequences for subsequent analyses. These primers were synthesized by Ruibiotech, Beijing, China. Details of the sequences of primers used are compiled in Table 1, highlighting the precision and methodological rigor applied in the quantification of miRNA expression.

**Table 1:** Sequences of the primers used for MicroRNA quantification

Name of Gene	Primer Sequence(5’-3’)
bta-miR-2887	F: CGCGGGACCGGGGTCC
	R: AGTGCAGGGTCCGAGGTATT
bta-miR-2892	F: GGCGACGGAGGCGCGA
	R: AGTGCAGGGTCCGAGGTATT
bta-miR-2424	F: CGCGCAGATCTTTGGTAATCT
	R: AGTGCAGGGTCCGAGGTATT
bta-miR-223	F: GCGCGTGTCAGTTTGTCAAAT
	R: AGTGCAGGGTCCGAGGTATT
bta-miR-135b	F: CGCGTATGGCTTTTCATTCCT
	R: AGTGCAGGGTCCGAGGTATT
bta-miR-2332	F: CGCGGTTTAAGGTCTTGGAG
	R: AGTGCAGGGTCCGAGGTATT
bta-miR-7	F: CGCGTGGAAGACTAGTGATTTT
	R: AGTGCAGGGTCCGAGGTATT
bta-miR-3600	F: CGCGACAGTTCTTCAACTGG
	R: AGTGCAGGGTCCGAGGTATT
bta-miR-378	F: CGCGACTGGACTTGGAGTCA
	R: AGTGCAGGGTCCGAGGTATT
bta-miR-221	F: CGCGAGCTACATTGTCTGCT
	R: AGTGCAGGGTCCGAGGTATT
bta-miR-222	F: GCGCGAGCTACATCTGGCTA
	R: AGTGCAGGGTCCGAGGTATT
bta-miR-26c	F: CGCGAGCCTATCCTGGATTA
	R: AGTGCAGGGTCCGAGGTATT
bta-miR-221 stem‒loop sequence	5’GTCGTATCCAGTGCAGGGTCCGAGGTATTCGCACTGGATACGACAAACCC-3’
GAPDH	F: TGCACCACCAACTGCTTAG
	R: GATGCAGGGATGATGTTC

### miRNA and plasmid constructs

The sequence corresponding to bta-miR-221 is denoted as 5’-AGCUACAUUGUCUGCUGGGGUUU-3’. Synthetic analogues of bta-miR-221, including mimics, a negative control (NC), an inhibitor, and an inhibitor NC, were custom-produced by Suzhou Genepharma Co., Shanghai, China. To explore the interaction between bta-miR-221 and its predicted target, the ATG7 3’UTR (388 bp) from bovine sources was amplified, cloned and inserted into the C-terminus of the firefly luciferase gene within the psi-check2 vector, resulting in the creation of a wild-type plasmid (ATG7-3’UTR-psi-check2-WT). This construct was further modified to produce a mutant version (ATG7-3’UTR-psi-check2-Mut), altering the bta-miR-221 binding site. Additionally, the coding sequence (CDS) region of bovine-derived ATG7 was amplified and integrated into the pmCherry-N1 vector to generate the mCherry-ATG7 fluorescent reporter plasmid. These plasmid constructs were meticulously prepared by GeneCreate Biological Engineering Co., Ltd, Wuhan, China. The pCMV-GFP-LC3B plasmid, which was used for visualizing autophagic activity, was obtained from Beyotime. This comprehensive set of molecular tools enabled a detailed analysis of the regulatory impact of bta-miR-221 on ATG7 expression and its subsequent effects on autophagic processes.

### miRNA target validation and dual-luciferase assays

The downstream targets of bta-miR-221 were predicted using the online databases TargetScan and miRBase, which are valuable resources for identifying potential miRNA binding sites and their corresponding target genes. For sequence alignments and comparisons, SnapGene software served as an essential tool, facilitating the detailed analysis of nucleotide sequences. To assess the expression levels of ATG7 mRNA within cells, qPCR was performed using specific primers: forward primer, 5’-GTTCCTCCTCTTGACATTTGC-3’; and reverse primer, 5’-TCTGACAAAGGGCATCATACG-3’. This technique enabled the quantification of ATG7 mRNA, providing insights into the regulatory effects of bta-miR-221.

To validate the predictions regarding bta-miR-221 downstream targets, HEK-293T cells were cultured in 24-well plates until they reached approximately 70% confluence. At this juncture, cells were transfected with either the ATG7-3’UTR-psi-check2-WT or ATG7-3’UTR-psi-check2-Mut plasmid in combination with bta-miR-221 mimics or inhibitors, as well as the corresponding control vectors. Twenty-four hours posttransfection, the cells were lysed and analysed for both firefly and Renilla luciferase activities using the Dual-Luciferase Reporter Gene Cell Lysate and Dual-Lumi™ II Dual-Luciferase Reporter Gene Detection Kit from Beyotime. This dual-reporter assay system allowed for the precise measurement of luciferase activity, thus confirming the influence of bta-miR-221 on the expression of the ATG7 gene via its 3’UTR, and thereby shedding light on the posttranscriptional regulatory mechanisms at play.

### Electron microscopy analysis

MDBK cells were inoculated with BVDV at a multiplicity of infection (MOI) of 1, while uninfected cells served as the control group. Following a 48-hour incubation period, the cells were washed with PBS at pH 7.4, and then fixed in situ with a fixative solution composed of 2.5% glutaraldehyde and 2% paraformaldehyde (PFA). After collection, the cells were postfixed with osmium tetroxide, followed by a graded dehydration process utilizing ethanol and acetone. The samples were then stained with uranyl acetate and lead citrate. Ultrastructural analysis, which focused on the identification of autophagosomes and autophagic lysosomes, was conducted using a Talos L120C transmission electron microscope (TEM) (Invitrogen, Carlsbad, CA, USA), which provided detailed insights into the cellular responses to BVDV infection at the ultrastructural level.

### Western blotting and immunoprecipitation

The cells were rinsed with prechilled PBS and lysed using RIPA lysis buffer (Solarbio, China) supplemented with a protease inhibitor cocktail at a 1:100 dilution. The protein concentrations in the lysates were quantified using the BCA Protein Assay Kit (Beyotime). The cell lysates were then subjected to sodium dodecyl sulphate‒polyacrylamide gel electrophoresis (SDS-PAGE) and subsequently transferred onto polyvinylidene difluoride (PVDF) membranes (Sigma‒Aldrich). The membranes were blocked at room temperature for 15 min with QuickBlock™ Blocking Buffer for Western blotting (Beyotime) and incubated overnight at 4 °C with primary antibodies against ATG7 (1:1000), LC3B (1:1000), SQSTM1/p62 (1:1000), GFP (1:1000), mCherry (1:1000), and GAPDH (1:50,000). Following primary antibody incubation, the membranes were incubated with the appropriate Dylight 800, goat anti-mouse IgG or Dylight 800, goat anti-rabbit IgG (1:20,000) for 1 h at room temperature in the dark, and then the protein bands were detected.

For immunoprecipitation, the membrane was lysed in Western and IP cell lysis solution (Beyotime) for 10 min, followed by centrifugation to collect the supernatant. The supernatant from the test group was then incubated with anti-GFP magnetic beads (Beyotime), while the control group’s supernatant was incubated with mouse IgG magnetic beads (Beyotime) for 2 h. After incubation, the supernatant of the test group was washed three times with Western and IP cell lysis solution, mixed with 1× SDS loading buffer, and boiled at 100 °C. The proteins were subsequently analysed by immunoblotting using mCherry and GFP antibodies, providing insights into protein-protein interactions and posttranslational modifications.

### Confocal microscopy techniques

Following the pretreatment procedure, the cells were washed three times with PBS before being fixed with 4% paraformaldehyde at room temperature for 10 min. After another round of washing, the cells were stained with 4’,6-diamidino-2-phenylindole (DAPI) staining solution (Solarbio) for 10 min to visualize the nuclei. The stained cells were then examined under a laser scanning microscope (Leica, Frankfurt, Germany), facilitating the detailed observation of nuclear morphology and the colocalization of target proteins through DAPI staining patterns.

### Statistical methodology

For each experiment conducted, three independent replicates were performed to ensure the reliability and reproducibility of the results. The data from these experiments are presented as the means ± standard deviations, providing a measure of variability and central tendency. Statistical analyses to evaluate the differences between groups were conducted using the t test function in GraphPad Prism software (version 8.0.2). A p value less than 0.05 was considered statistically significant, indicating a meaningful difference between the compared datasets. This rigorous statistical approach underlines the commitment to accuracy and the scientific validity of the findings.

## Results

### Bta-miR-221 expression in BVDV-infected cells

**Fig. 2:**
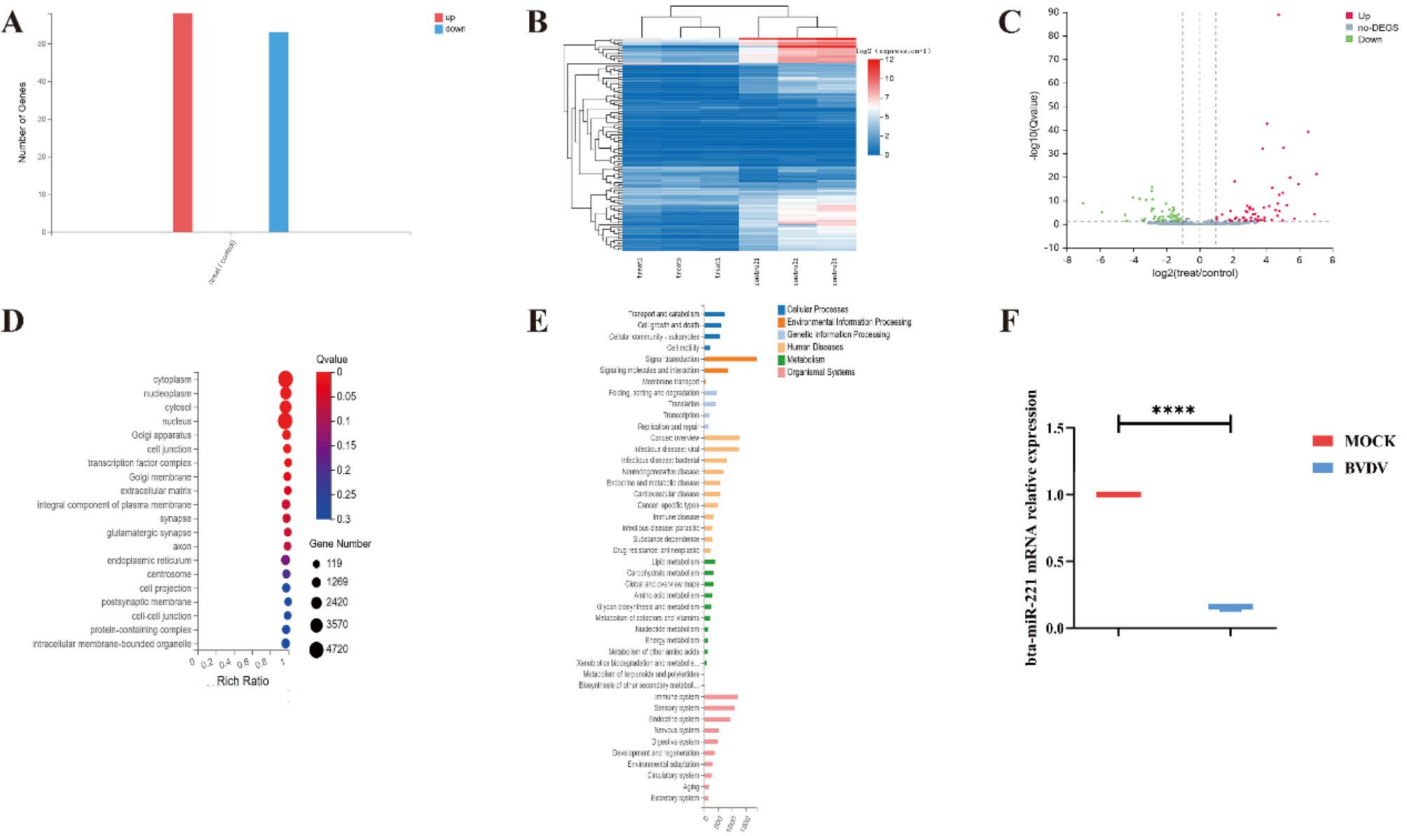
bta-miR-221 Expression Dynamics in BVDV-Infected Cells. Cells infected with BVDV at a multiplicity of infection (MOI) of 1 underwent miRNA library construction for detailed expression analysis. **A** and **B**, Clustering heatmaps showing the differential expression of miRNAs; a gradient from red to blue indicates expression levels from high to low, respectively. **C**, Differential expression volcano plot for miRNAs. **D**, Gene Ontology (GO) analysis results for commonly differentially expressed miRNAs. E, Kyoto Encyclopaedia of Genes and Genomes (KEGG) pathway analysis of these miRNAs. F, Quantification of the relative expression of bta-miR-221

In this study, we identified 696 miRNAs across all samples via the BGI miRNA library construction strategy. Among these miRNAs, 101exhibited significant differential expression: 58 were upregulated, and 53 were downregulated (Fig. 2-A). We assessed the variations in these differential miRNAs through clustering heatmaps and volcano plots, revealing their differential expression profiles (Fig. 2-B and C). Gene Ontology (GO) analysis revealed that these miRNAs played roles in several crucial cellular processes, including plasma membrane synthesis and protein synthesis (Fig. 2-D). Additionally, KEGG pathway analysis linked these miRNAs to key biological pathways, such as the immune pathway and the MAPK signalling pathway (Fig. 2-E). We selected twelve miRNAs with significant differential expression for validation via quantitative PCR (qPCR). Notably, bta-miR-221 expression markedly changed (Fig. 2-F), confirming its role as the focus of our subsequent experiments.

### The role of Bta-miR-221 in suppressing BVDV replication

**Fig. 3:**
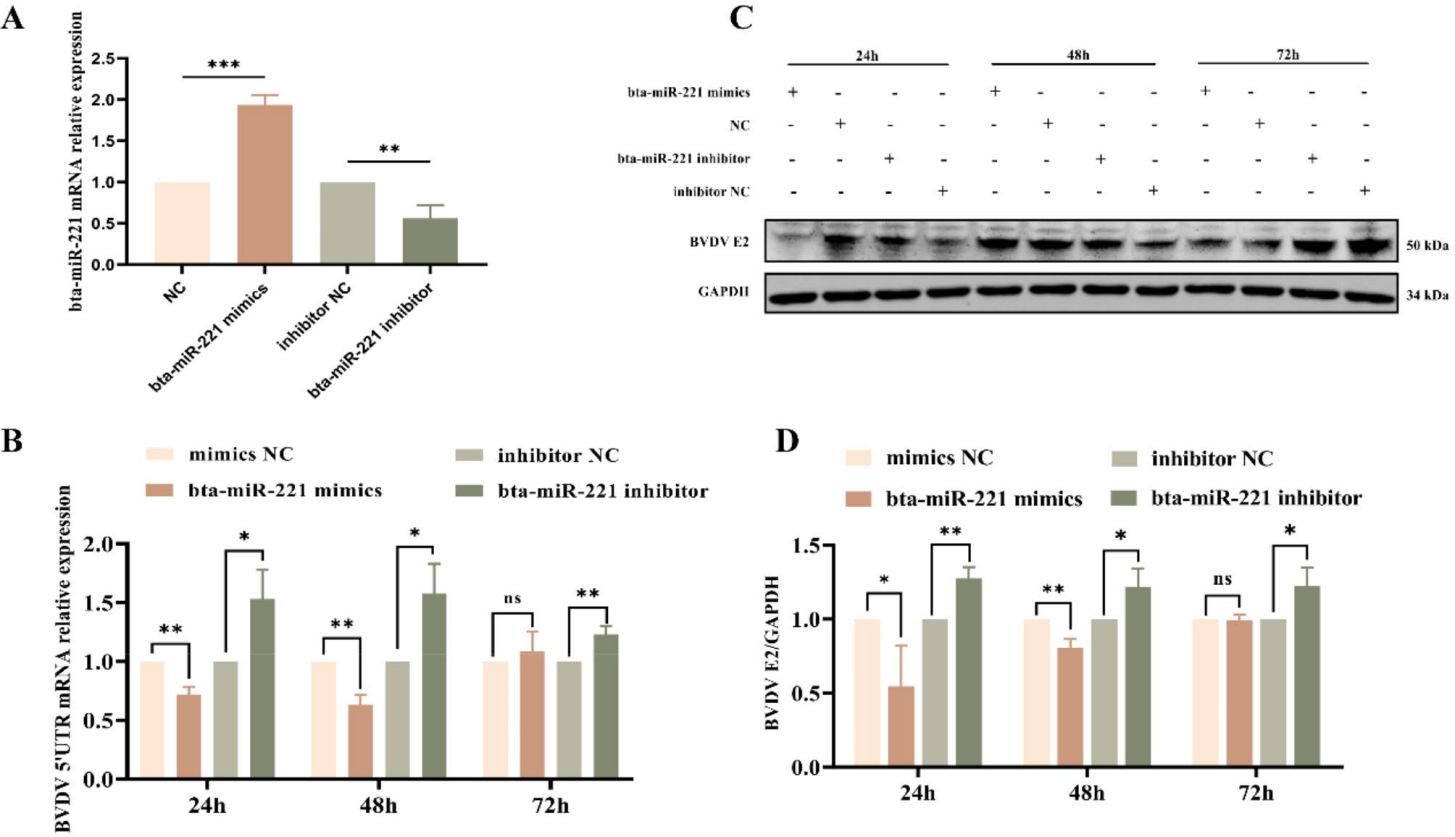
Suppression of BVDV Replication by bta-miR-221 Mimics. **A**, Transfection of MDBK cells with bta-miR-221 mimics and inhibitors; the transfection efficiency was assessed via quantitative PCR (qPCR) at 12 h posttransfection. **B**, qPCR analysis of BVDV 5’UTR mRNA levels across different treatment groups, indicating the impact of bta-miR-221 modulation on viral mRNA expression. **C** and **D**, Western blot analyses evaluating the variations in BVDV E2 protein levels among the treatment groups

In this study, we observed a reduction in bta-miR-221 expression in BVDV-infected cells. Drawing upon existing research linking microRNAs to viral replication dynamics [24], we hypothesized that bta-miR-221 might influence BVDV replication. To investigate this hypothesis, we transfected MDBK cells with bta-miR-221 mimics and inhibitors for 12 h, which led to a significant alteration in bta-miR-221 levels: the mimics upregulated bta-miR-221 expression, whereas the inhibitors downregulated it (Fig. 3-A). Subsequent infection of these cells with BVDV at an MOI of 1 allowed the assessment of intracellular BVDV 5’UTR mRNA expression and BVDV E2 protein levels at 24, 48, and 72 h post infection (Fig. 3-B, C, and D). Notably, the bta-miR-‒21 mimic group presented a significant reduction in BVDV 5’UTR mRNA expression at 24 and 48 h post infection, with corresponding decreases in BVDV E2 protein levels. However, by 72 h, these differences were no longer statistically significant. Conversely, cells treated with the bta-miR-221 inhibitor presented significant increases in both BVDV 5’UTR mRNA expression and BVDV E2 protein levels at all the observed time points compared with those in the inhibitor negative control (NC) group. These results suggested that the modulatory effect of bta-miR-221 on BVDV replication within MDBK cells was time dependent, with the most pronounced effect observed within the first 48 h post infection.

### Targeting of the autophagy gene ATG7 by Bta-miR-221

**Fig. 4:**
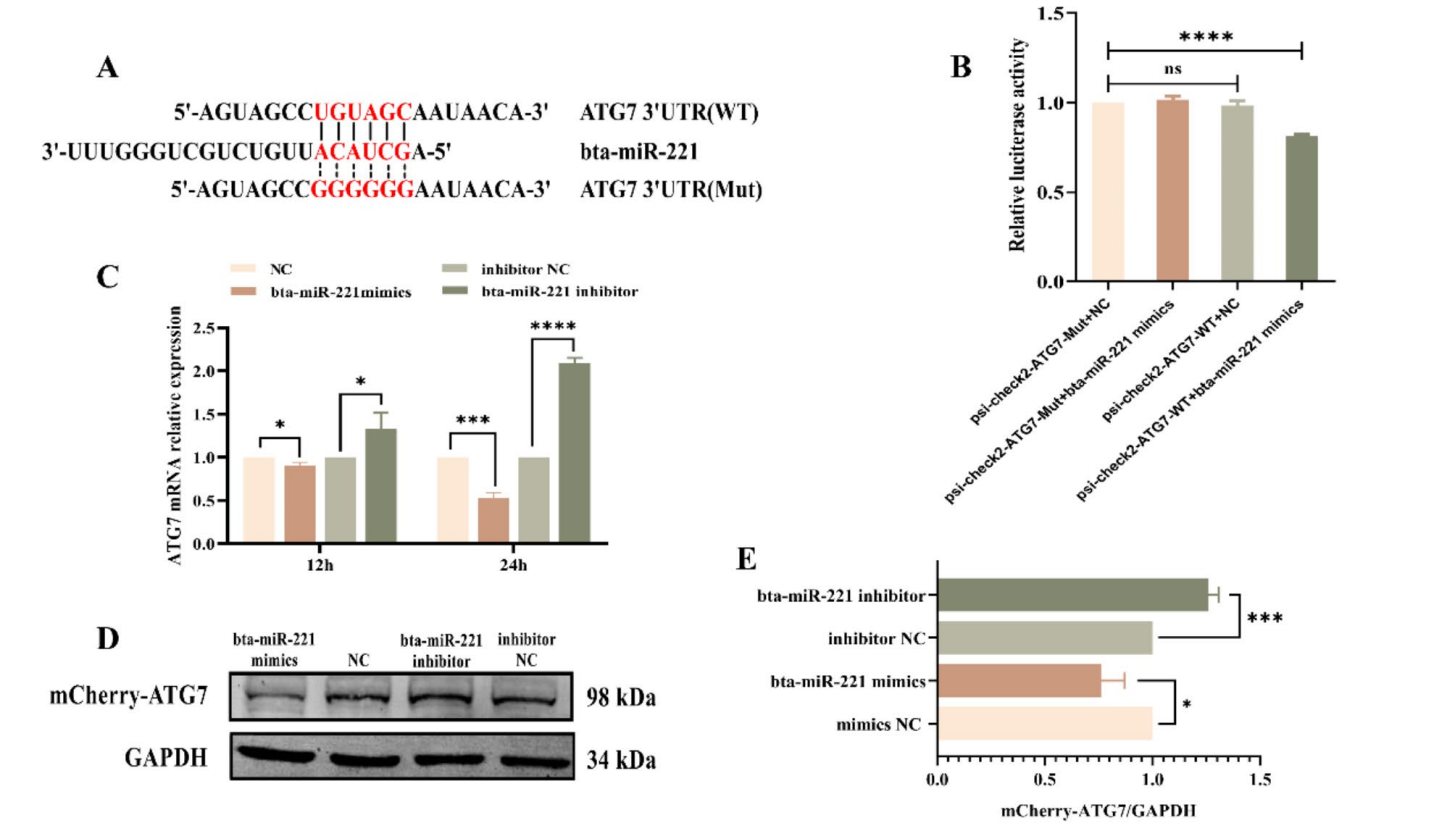
bta-miR-221 Targets and Suppresses ATG7 Expression. **A**, bioinformatics tools were used to predict the interaction site between bta-miR-221 and the ATG7 3’UTR. **B**, Effect of bta-miR-221 on ATG7 expression. HEK-293T cells were cotransfected with either wild-type or mutant ATG7-3’UTR-psiCHECK2 plasmids along with bta-miR-221 mimics or inhibitors, followed by luciferase assay measurements 24 h posttransfection to assess regulatory impacts. **C**, Details of the experiment in MDBK cells, where transfection with bta-miR-221 mimics or inhibitors (along with their respective negative controls) preceded qPCR analysis at 12 and 24 h to quantify ATG7 mRNA levels. **D** and **E**, The influence of bta-miR-221 on ATG7 protein levels was tracked, and the intracellular concentration of mCherry-ATG7 protein in HEK-293T cells 24 h after cotransfection with bta-miR-221 mimics or inhibitors was determined

Bioinformatics analyses revealed that ATG7 was a downstream target gene of bta-miR-221that bound to the 3’UTR of ATG7 through covalent interactions (Fig. 4-A). To validate this prediction, we conducted a dual-luciferase reporter assay. The results revealed that bta-miR-221 mimics significantly decreased the luciferase activity of the wild-type ATG7-3’UTR construct (ATG7-3’UTR-psiCHECK2-WT), whereas the mutant construct (ATG7-3’UTR-psiCHECK2-Mut) remained unaffected, confirming the specificity of the interaction (Fig. 4-B). To further investigate the functional impact of this interaction, we examined the endogenous expression levels of ATG7 mRNA in MDBK cells. Transfection with bta-miR-221 mimics for 12 and 24 h led to a significant reduction in ATG7 mRNA levels, with the effect being more pronounced at 24 h. Conversely, the bta-miR-221 inhibitor significantly increased ATG7 mRNA expression at both time points, underscoring the regulatory effect of bta-miR-221 on ATG7 expression (Fig. 4-C). Protein expression analyses via Western blot corroborated these findings: bta-miR-221 mimics substantially reduced the expression of the exogenous mCherry-ATG7 fusion protein, whereas inhibition of bta-miR-221 resulted in a notable increase in mCherry-ATG7 protein levels (Fig. 4-D and E). Collectively, these results demonstrated a direct and negative regulatory relationship between bta-miR-221 and ATG7, which was mediated through the 3’UTR of ATG7, with significant implications for the understanding of the molecular dynamics influenced by bta-miR-221.

### Autophagy induction by BVDV in MDBK cells

**Fig. 5:**
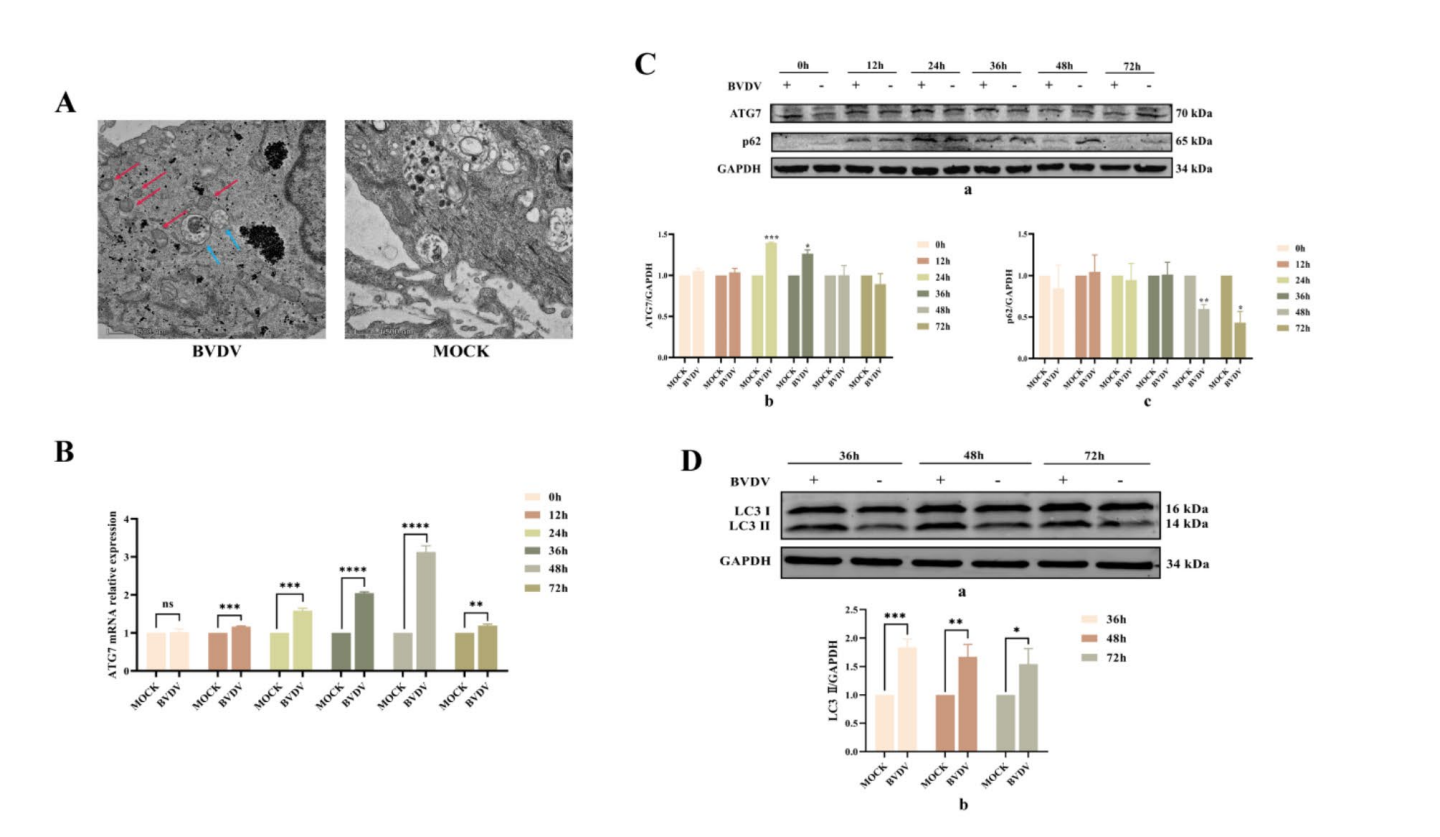
Autophagy induction in BVDV-infected MDBK cells. **A**, Autophagic response in MDBK cells at 48 h post infection with BVDV at a multiplicity of infection (MOI) of 1, as visualized via transmission electron microscopy. Autophagic lysosomes (indicated by blue arrows) and autophagosomes (red arrows) were markedly more prevalent in the BVDV-infected (experimental) group than in the MOCK (control) group. **B**, Temporal analysis of ATG7 mRNA expression levels following BVDV infection, as quantified via qPCR. **C** and **D**, Details of the protein expression dynamics of p62, ATG7, and LC3 during the course of BVDV infection, as determined by Western blotting

To elucidate the relationship between BVDV infection and autophagy in MDBK cells, we first utilized transmission electron microscopy to observe autophagic structures. We detected a significant increase in the number of autophagic vesicles, characterized by their double-membrane structure, and autolysosomes, characterized by their single-membrane structure, in BVDV-infected cells compared with controls cells cultured in DMEM (Fig. 5-A). These findings suggested that BVDV infection triggered autophagy in MDBK cells. Subsequently, we assessed the expression of autophagy-related genes and proteins following BVDV infection. Quantitative PCR (qPCR) analysis revealed a significant upregulation of ATG7 mRNA expression at 36 and 48 h post infection (Fig. 5-B). Consistent with these findings, Western blot analysis revealed a progressive increase in ATG7 protein expression, which commenced at 24 h and peaked at 36 h post infection. Concurrently, a significant reduction in the levels of the autophagy substrate protein p62 was observed at 48 and 72 h post infection. This temporal pattern of protein expression changes suggested an increase in autophagic flux in response to infection (Fig. 5-C). Further exploration of the impact of BVDV on cellular autophagy included an analysis of protein levels of LC3, a key autophagy marker, after 36, 48, and 72 h of infection. The ratio of the lipidated form of LC3-II to its precursor LC3-I exceeded 1 at all the observed time points (Fig. 5-D), confirming that autophagy was induced in a time-dependent manner post BVDV infection. These results revealed a significant modulation of key autophagy markers, further confirming the autophagic response to BVDV infection and the changes in autophagy-related gene expression over time. In summary, our findings demonstrated that BVDV infection stimulated autophagy in MDBK cells, as evidenced by increased numbers of autophagic vesicles, increased ATG7 mRNA and protein expression, reduced p62 levels, and elevated LC3-II/LC3-I ratios. These results highlighted a significant cellular response to viral infection, suggesting that autophagy might play a critical role in the defence mechanism of MDBK cells against BVDV.

### The Bta-miR-221 mediated ATG7-LC3 autophagy pathway

**Fig. 6:**
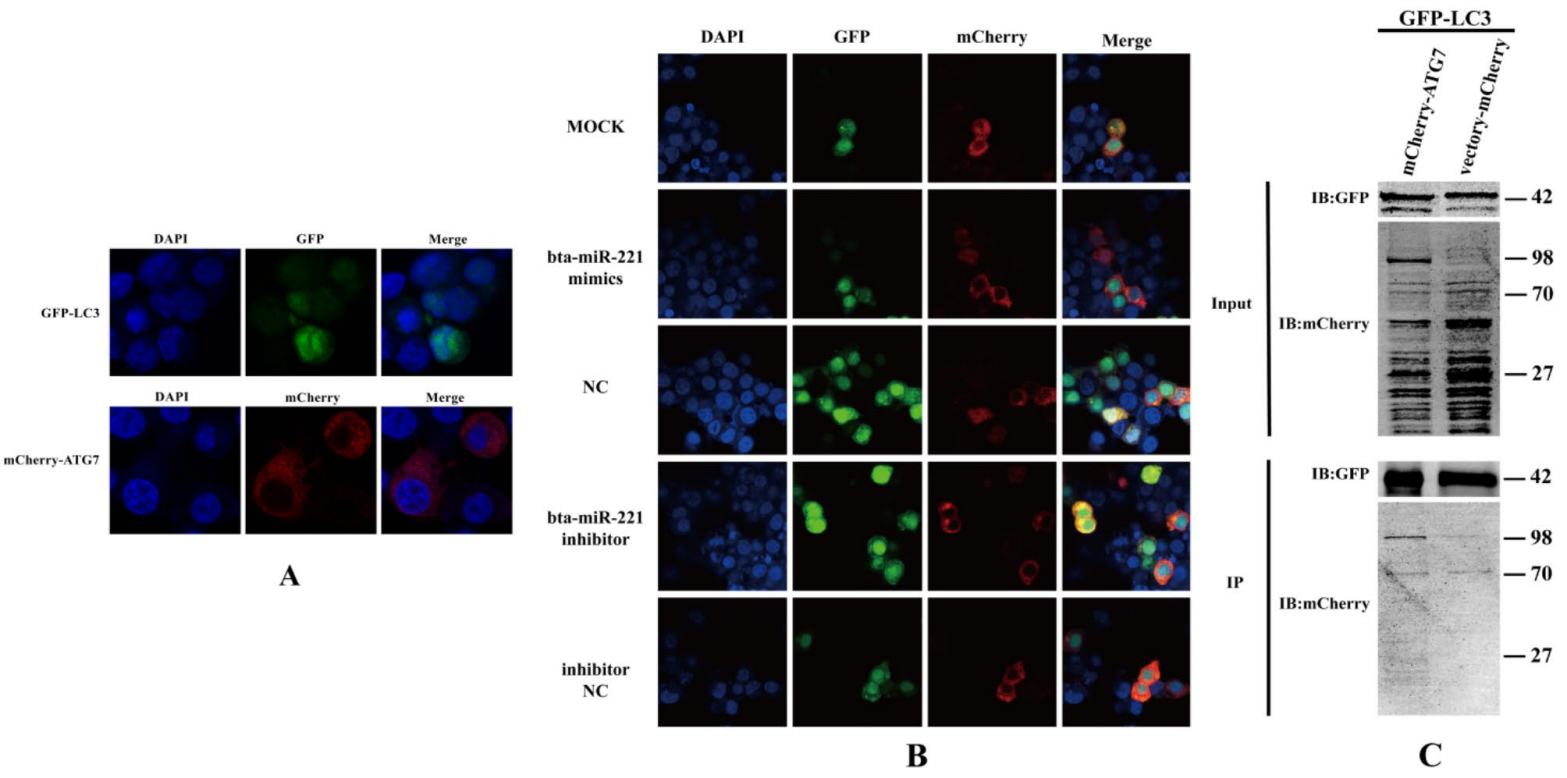
Modulation of the ATG7-LC3 Autophagy Pathway by bta-miR-221 Mimics. **A**, The initial localization of GFP-LC3 and mCherry-ATG7 in 293T cells was observed via confocal microscopy, establishing baseline cellular distribution patterns of these autophagy markers. **B**, Examination of the impact of bta-miR-221 mimics and inhibitors on the autophagy pathway. The bta-miR-221 mimics or inhibitors (and their respective controls) were cotransfected with GFP-LC3 and mCherry-ATG7 in HEK-293T cells. Cellular localization and interaction patterns were observed through confocal microscopy imaging 24 h posttransfection. **C**, HEK-293T cells were transfected with combinations of GFP-LC3 + mCherry-ATG7 and GFP-LC3 + vector-mCherry, followed by immunoprecipitation to analyse the association between the GFP and mCherry proteins, as quantified by Western blotting

ATG7, an E1-like activating enzyme, plays a pivotal role in autophagy initiation, catalysing the conversion of LC3I to LC3II in concert with ATG3, marking the commencement of autophagosome formation [25]. Using laser confocal microscopy, we observed differential localization patterns of these autophagy-related proteins within cells; LC3 predominantly resided within the nucleus, whereas ATG7 was localized in the cytoplasm (Fig. 6-A). Interestingly, upon cotransfection, LC3 and ATG7 exhibited a significant degree of colocalization within the cytoplasm, suggesting that their interacted during the autophagic process. To further investigate the regulatory effect of bta-miR-221 on the ATG7-LC3 interaction, 293T cells expressing both LC3 and ATG7 were transfected with bta-miR-221 mimics or inhibitors. The results demonstrated that the bta-miR-221 mimic group presented diminished colocalization of LC3 and ATG7, suggesting an inhibitory effect on their interaction, whereas the bta-miR-221 inhibitor group presented increased colocalization, suggesting a promoting effect on the autophagic pathway (Fig. 6-B). This interaction between mCherry-ATG7 and GFP-LC3 was further validated through immunoprecipitation experiments, confirming the physical interaction between ATG7 and LC3 (Fig. 6-C). These findings collectively substantiated the modulatory role of bta-miR-221 in the ATG7-LC3 autophagy pathway. Our study revealed that bta-miR-221 inhibited autophagy in MDBK cells by impacting the pivotal ATG7-LC3 interaction, providing insight into the molecular mechanisms underlying the autophagic process and its modulation by microRNAs. In summary, this investigation not only elucidated the bta-miR-221-mediated regulation of the ATG7-LC3 pathway, which is critical for autophagy initiation, but also highlighted the broader implications of miRNA involvement in autophagic regulation, suggesting potential avenues for therapeutic intervention in diseases where autophagy plays a crucial role.

## Discussion

BVDV significantly impacts the livestock industry, with persistently infected (PI) cattle leading to substantial economic losses. Despite this economic impact, preventive and therapeutic measures against BVDV remain limited. Our study aimed to elucidate the pathway of BVDV replication and identify related targets to innovate prevention and treatment strategies for BVDV. We discovered that BVDV infection suppressed bta-miR-221 expression, thereby promoting the ATG7-LC3 autophagy pathway and inducing cellular autophagy [26]. This process facilitated BVDV replication, underscoring the pivotal role of bta-miR-221’s pivotal role in the viral life cycle.

Viruses exploit various cellular factors and signalling pathways to increase their replication, whereas host organisms deploy numerous defence mechanisms to maintain homeostasis [27]. Understanding these virus-host interaction pathways is crucial for developing viral therapies and discovering antiviral drugs. In our investigation, miRNA library construction revealed a significant reduction in bta-miR-221 expression in BVDV-infected cells. Since the discovery of microRNAs (miRNAs) in the nematode *Caenorhabditis elegans* in the 1990s [28], extensive research has shown that viruses and hosts can regulate gene expression through miRNAs, thereby controlling viral replication [29, 30]. Notably, Tong-Qing An et al. (2020) identified multiple miRNAs that influence PRRSV replication and their mechanisms [31]. Similarly, BVDV, which belongs to the same genus as HCV in the Flaviviridae family, serves as an alternative model for HCV studies [32]. Eun Byul Lee et al. (2020) demonstrated that miR-99a regulates mTOR/SREBP-1c, reducing intracellular lipid accumulation and HCV replication [33]. Recent studies have substantiated the significant role of microRNAs (miRNAs) in the replication process of BVDV. Specifically, bta-miR-2383 has been demonstrated to regulate BVDV replication through the lnc-CYLD/miR-2383/CYLD axis, which represents a complex regulatory network [34]. Additionally, bta-miR-2411 modulates the expression of the Pelota gene, thereby influencing the replication dynamics of BVDV [35]. Furthermore, bta-miR-29b has been shown to regulate apoptosis by directly targeting caspase-7 and NAIF1, thereby inhibiting viral replication within MDBK cells [36]. These findings underscore the intricate interplay between miRNAs and viral processes, offering novel insights into potential therapeutic targets for controlling BVDV infection. Our findings contribute to this body of work by demonstrating a novel role for bta-miR-221 in regulating BVDV replication. Over the past decade, miR-221 has attracted considerable interest across numerous scientific studies. The oncogenic properties of miRNAs have revealed their capacity to exert significant influence within the tumour microenvironment [37]. Concurrently, in the realm of virology, miR-211 has been identified as a pivotal host element facilitating the replication of hepatitis C virus (HCV). Building upon our previous observations that underscore the relevance of a shared research focus for both HCV and BVDV, this study delves deeper into the intricate relationship between miR-221 and BVDV, elucidating potential intersections in their biological roles [38]. The observed reduction in bta-miR-221 expression in BVDV-infected MDBK cells not only highlights a potential viral survival strategy but also reveals bta-miR-221 as a key player in the host antiviral defence mechanism. These findings open new pathways for exploring miRNA‒based therapeutics, offering a promising direction for the prevention and treatment of BVDV infections.

The involvement of host miRNAs in regulating organismal life processes, including cellular autophagy and apoptosis, has garnered increasing attention. Notably, the miR454-FAM83A-TSPAN1 axis, with TSPAN1 acting as a positive autophagy regulator and a target of microRNA454, has been shown to facilitate autophagy and influence pancreatic cancer cell proliferation [39]. Similarly, the absence of miR-33 in macrophages has been associated with increased mitochondrial homeostasis and increased autophagy [40], underscoring the diverse roles of miRNAs in cellular function and disease pathology. In the present study, we extended these insights by demonstrating that bta-miR-221 directly targeted the 3’UTR terminus of ATG7, a gene that is pivotal for autophagy initiation. This interaction resulted in the suppression of ATG7 expression, both endogenously within cells and exogenously in our model systems. Furthermore, we elucidated the role of bta-miR-221 in modulating the classical ATG7-LC3 autophagy pathway in MDBK cells. Our findings indicated that bta-miR-221 acted as a regulatory node within this pathway, influencing autophagy through its interaction with ATG7. This study contributes to the growing body of evidence that host miRNAs are integral to the regulation of critical cellular processes. By identifying bta-miR-221-mediated inhibition of ATG7 and its consequent effects on the ATG7-LC3 pathway, we provide new insights into the complex interplay between miRNAs and autophagy. These findings not only enhance our understanding of miRNA function in cellular regulation but also open potential avenues for therapeutic intervention in diseases involving autophagy dysregulation.

Autophagy, a critical cellular degradation mechanism, is essential for maintaining cellular homeostasis by removing intracellular waste and pathogens [41, 42]. This process has been implicated in the life cycle of various viruses, including HCV, which utilizes host cell autophagy for its replication, maturation, and release [43].In addition, in 2014, Fu et al. reported that the BVDV envelope proteins Erns and E2 are instrumental in modulating autophagy through the enhancement of autophagosome formation [44]. In 2017, Rajput et al. reported that both cytopathic and noncytopathic BVDV strains elicit autophagy and are capable of replication within Madin–Darby bovine kidney (MDBK) and primary bovine turbinate (Bt) cells [45]. In 2023, Seung-Uk et al. reported that noncytopathic (ncp) bovine viral diarrhoea virus genotype 2 (BVDV2) enhances viral replication by inducing cellular autophagy, as evidenced by the promotion of the conversion of LC3-I to LC3-II, the upregulation of ATG5 and Beclin1, and the degradation of p62/SQSTM1 [46].Wang et al. reported that these two BVDV biotypes employ different regulatory strategies to regulate the unfolded protein response (UPR) and ER stress-mediated autophagy pathways, highlighting the intricate interplay between viral mechanisms and cellular homeostasis [47]. Our study built upon these findings, demonstrating that BVDV infection triggered a similar autophagic response in MDBK cells. Through detailed transmission electron microscopy, we observed an abundance of autophagic vesicles and lysosomes in BVDV-infected cells. Western blot analysis further confirmed the induction of autophagy by BVDV, as evidenced by the conversion of LC3-I to LC3-II, increased expression of ATG7, and degradation of p62/SQSTM1. Importantly, our research highlighted the role of bta-miR-221 in this process. We found that BVDV modulated MDBK autophagy via the bta-miR-221-mediated ATG7-LC3 pathway, with bta-miR-221 playing a pivotal role in inhibiting BVDV replication. This multifaceted approach delineated the inhibitory effects of bta-miR-221 mimics on the ATG7-LC3-mediated autophagy pathway, providing insight into the molecular dynamics that regulated autophagy.

**Fig. 7:**
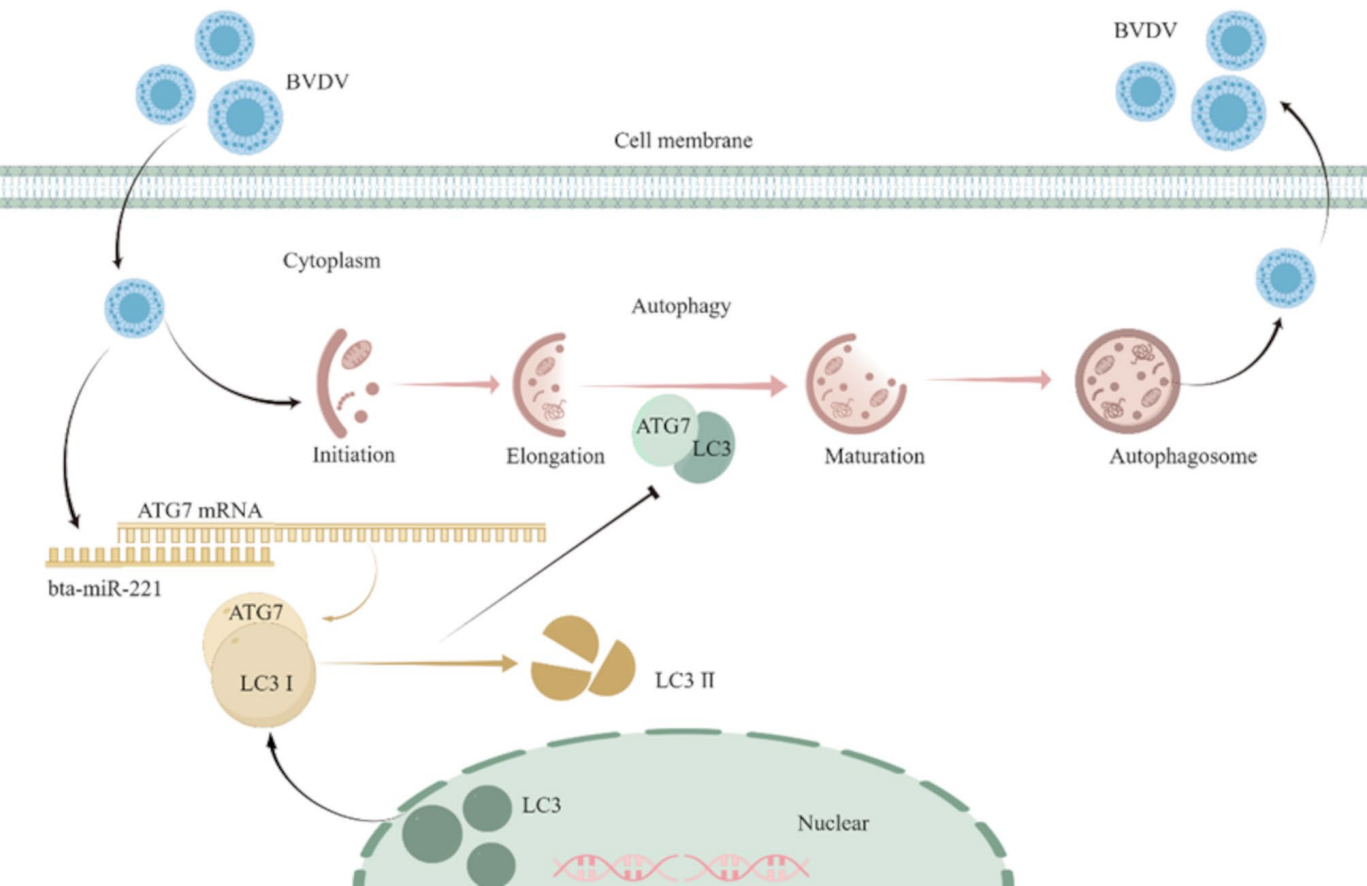
Mechanistic Model of the role of bta-miR-221 in Inhibiting BVDV Replication. This model illustrates the dual role of bta-miR-221 in the cellular response to BVDV infection. BVDV infection leads to a decrease in bta-miR-221 expression, consequently triggering the activation of the ATG7-LC3 autophagy pathway. This autophagic response potentially aids in the viral replication process. Conversely, bta-miR-221 inhibits BVDV replication within the cell, revealing a complex interplay among host miRNA regulation, autophagy, and viral replication dynamics. The diagram was created using FigDraw (https://www.figdraw.com/), providing a visual summary of the inhibitory mechanism of bta-miR-221 on BVDV replication through the modulation of cellular autophagy pathways

In conclusion, this study not only confirmed the capacity of BVDV to regulate autophagy in MDBK cells, but it also elucidated the underlying molecular mechanism, involving bta-miR-221-mediated modulation of the ATG7-LC3 pathway (Fig. 7). These findings provide a deeper understanding of the complex interplay between viral pathogens and host cellular processes, offering new perspectives on the role of miRNAs in viral replication. By shedding light on the involvement of bta-miR-221 in modulating autophagy and inhibiting BVDV replication, our research opens potential avenues for therapeutic interventions targeting autophagy-related pathways in viral diseases.

## Conclusion

In summary, our comprehensive analysis revealed a novel mechanism by which BVDV manipulated host cellular processes to facilitate its replication. We discovered that BVDV infection led to the downregulation of bta-miR-221, a critical regulatory miRNA within the host cells. This reduction in bta-miR-221 expression paved the way for activation of the ATG7-LC3 autophagy pathway, a key process for maintaining cellular homeostasis. Intriguingly, while BVDV induced autophagy to benefit its replication cycle, bta-miR-221 emerged as a pivotal antagonist of this viral strategy by inhibiting the replication process of BVDV.

Our findings not only shed light on the sophisticated interplay between BVDV and the autophagic machinery of the host, but the also highlight the potential of targeting bta-miR-221 and the ATG7-LC3 pathway as novel therapeutic avenues for controlling BVDV infection. By revealing the role of bta-miR-221 in modulating autophagy and its impact on viral replication, this study contributes valuable insights into the molecular mechanisms of BVDV pathogenesis and opens new pathways for the development of antiviral strategies.

## Data Availability

Not applicable.

## Abbreviations

BVD: Bovine viral diarrhoea
BVDV: Bovine viral diarrhoea virus
MDBK: Madin‒Darby bovine kidney
293T: Human embryonic kidney 293T cells
PI: Persistent Infections
ATG: Autophagy-Related Genes
PE: Phosphatidylethanolamine
Ncp: Noncytopathic
Cp: Cytopathic
